# Comments on “Low-Cost Alternative External Rotation Shoulder Brace and Review of Treatment in Acute Shoulder Dislocations”

**DOI:** 10.5811/westjem.2015.4.26247

**Published:** 2015-04-29

**Authors:** Kyle Lacy, Chris Cooke, Pat Cooke, Justin Schupach, Jon Carlson, Rahul Vaidya

**Affiliations:** Detroit Medical Center, Department of Orthopaedic Surgery, Detroit, Michigan; Detroit Medical Center, Department of Orthopaedic Surgery, Detroit, Michigan; Detroit Medical Center, Department of Orthopaedic Surgery, Detroit, Michigan; Wayne State University, Detroit Medical Center, Detroit, Michigan; Detroit Medical Center, Department of Orthopaedic Surgery, Detroit, Michigan; Detroit Medical Center, Department of Orthopaedic Surgery, Detroit, Michigan

In Reply:

We would like to thank the editors of the *Western Journal of Emergency Medicine* for the opportunity to reply to the letter to the editor by Jordan et al. regarding our paper “Low-Cost Alternative External Rotation Shoulder Brace and Review of Treatment in Acute Shoulder Dislocations.”^1^

Jordan et al. comment in their letter to the editor,^2^ “We feel that the narrative review in this publication does not provide a balanced overview of the clinical studies available and we question the value of external rotation in the management of these patients. They further state that, the paper is “likely to be subject to reporting bias.” Jordan et al.^2^ quote articles that question the value of external rotation bracing over internal rotation bracing for acute anterior dislocations.^3^–^7^ Each one of these publications^3^–^7^ is also referenced in our review^1^ and are the reason we clearly state in our article that “Posterior dislocations are immobilized in external rotation or a ‘gunslinger’ position of neutral rotation, abduction, and slight flexion.^8^ The position of immobilization for anterior shoulder dislocations is somewhat controversial,” and we repeat that “larger randomized controlled trials, as well as meta-analyses comparing external and internal rotation immobilization for acute traumatic anterior shoulder dislocation, have not shown a statistically significant difference in regards to recurrence of dislocation.”^3^–^7^

Jordan et al.^2^ further state that “a recent systematic review and meta-analysis also concluded that external rotation bracing for anterior shoulder dislocations is not advantageous.”^6^,^7^ Unfortunately they misquote Patterson et al.,^6^ which states in its conclusion, “Bracing in external rotation may provide a clinically important benefit over traditional sling immobilization, but the difference in recurrence rates did not achieve significance with the numbers available.”

Also in a commentary written by Bruce S. Miller on this article^8^ in the same journal, Miller questions the discrepant findings in this emerging body of evidence for external rotation bracing in anterior shoulder dislocations and feels it certainly warrants further investigation but does not discount the work of Itoi et al.^3^ as does our current letter to the editor.

Jordan et al.^2^ state, “The image of the sling provided demonstrates that this device produces only a small degree of external rotation,” and also state that “the clinical studies previously discussed^3^–^5^,^10^,^11^ only achieved 10 to 20 degrees of rotation and the illustration of the described technique in this paper suggests even less was achieved with this alternative brace.” Allow us to provide you with some better images (Figure) and mention that the degree of external rotation brace is adjustable depending on the amount of padding used in the bump. The placement of more padding anteriorly in the bump will create a greater degree of external rotation. The padding within the bump can also be compressed posteriorly to create a wedge shape, which aids in achieving additional external rotation.

Jordan et al. further comment that an additional factor not addressed is the acceptability of the splint to patients. “External rotation bracing is extremely inconvenient and poorly tolerated. Its prescription is associated with poor compliance which may limit its effectiveness.”^3^,^4^,^11^ Unfortunately the above statement is referenced by three randomized controlled trials that report the following compliance with bracing:

3) The external rotation brace was used in 27 patients, all but one of whom complied fully with the treatment. An internal rotation brace was used in 24 patients, all of whom complied with the treatment regime.^3^
4) The compliance rate with the immobilization was 47.4% (45 of 95) in the internal rotation group and 67.7% (63 of 93)) in the external rotation group.^4^
11) The compliance rate was 39 (53%) of 74 in the internal rotation group and 61 (72%) of 85 in the external rotation group (p=0.013).^11^

Our patients also seem to tolerate this soft padded brace pretty well.

In conclusion, we would like to reiterate that our low-cost brace is a good option for patients who would benefit from external rotation bracing of the shoulder or humerus. It can be adjusted to get up to 20 degrees of external rotation, and like other external rotation braces the compliance of use is very similar to internal rotation bracing. It is beneficial in posterior dislocations, certain humerus fractures, and for post-op care, and although the literature is controversial it may be an option for acute anterior shoulder dislocations. So we feel that our review is balanced not biased, represents the opinion of recent publications and feel that the letter to the editor misrepresents the literature as we have stated.

## Figures and Tables

**Figure:**
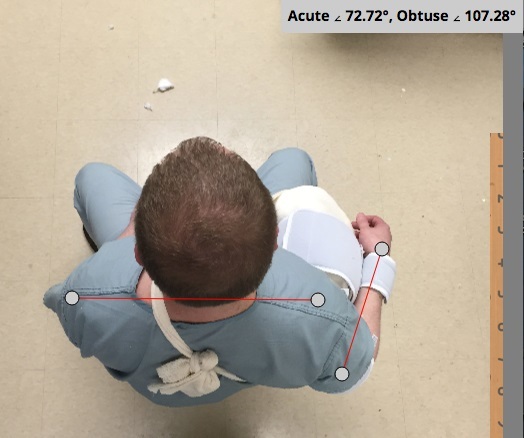
An overhead picture of the low-cost alternative external rotation shoulder brace demonstrating 18 degrees of external shoulder rotation. With additional padding in the bump anteriorly, greater external rotation can be achieved.
